# Short-term effects of two types of goggles on intraocular pressure and anterior eye segment biometrics

**DOI:** 10.1186/s12886-022-02308-y

**Published:** 2022-02-12

**Authors:** Xu Zhang, Huixian Wang, Yuan Nie, Wenjing Li

**Affiliations:** Army Eye Center, Xinjiang Military General Hospital, Beijing Road 754, 830000 Urimqi, China

**Keywords:** Intraocular pressure;central anterior chamber depth;anterior chamber volume;anterior chamber angle

## Abstract

**Background:**

To evaluate and compare the changes in intraocular pressure and anterior eye segment biometrics during and after wearing two types of commonly used swimming goggles.

**Methods:**

In a cross-sectional study, a total of 40 healthy adults aged between 18 and 60 years old were selected to wear two kinds of common swimming goggles (ocular socket and orbital goggles). Intraocular pressure and anterior segment biometry were evaluated before wearing, at 2 and 5 min of wearing, and at 5 min after removing the goggles. Intraocular pressure (IOP), corneal front keratometry values (K1, K2, Km), central corneal thickness (CCT), central anterior chamber depth (ACD), anterior chamber volume (ACV), and anterior chamber angle (ACA) were measured.

**Results:**

The IOP at 2 min (21.0 ± 2.2 mmHg) and 5 min (21.2 ± 2.3 mmHg) was significantly higher than before wearing goggles (17.7 ± 2.1 mmHg). The IOP after the goggles were removed and at 5 min after the goggles were removed was 18.4 ± 2.3 mmHg and 17.7 ± 2.1 mmHg, respectively. ACV, ACD, and ACA values all decreased while the googles were worn. After the goggles were removed, these changes gradually returned to baseline values, with no significant difference in the values before and after.

**Conclusion:**

This study proves that wearing orbital goggles can lead to an acute increase in IOP and a slight decrease in ACV, ACD, and ACA. However, once the goggles are removed, these indicators return to baseline levels, showing that wearing orbital goggles has no significant lasting effect on IOP and anterior segment parameters.

**Supplementary Information:**

The online version contains supplementary material available at 10.1186/s12886-022-02308-y.

## Introduction

Swimming is a sport for all ages, and its unique charm makes it one of the most popular fitness activities. Swimmers usually wear swimming goggles (SG) to prevent water from entering their eyes and to improve underwater visibility. The SG currently found on the market mainly come in two kinds: sport eye-socket goggles with a large frame and recreational eye-socket goggles with a small frame. Recent studies have shown that when goggles are worn, there is a brief increase in intraocular pressure (IOP) which returns to within the normal range once the goggles are removed [1–3].

However, until now, few studies have evaluated the changes in the biological parameters of the anterior segment while wearing SG, and there are no studies on the effects of different types of SG. As the increase of IOP may be related to the type of SG and the contact area between the goggles and the orbital tissues around the eye, [3, 4] this study aims to investigate whether differences exist in the short-term effects of wearing two types of SG with regard to the measurement of the anterior segment and IOP. Whether these changes are reversible after the goggles are removed is also investigated.

### Objective and methods

#### Objective

In this cross-sectional study, a total of 40 healthy Han Chinese men and women (22 females, 18 males) aged 18 to 60 years (mean age ± SD, 28.7 ± 7.6 years) were selected. All participants met the following inclusion criteria: (1) no history of refractive surgery or orthokeratology lens wear; (2) no general or ocular disease, and; (3) no current medications. The following exclusion criteria were used: refractive error (equivalent spherical lens) greater than ±6d, and physical and cognitive understanding ability not sufficient to complete the test. This study was approved by the Ethics Committee of the General Hospital of Xinjiang Military Region and carried out in accordance with the Declaration of Helsinki. All participants signed an informed consent form prior to the study.

### Methods

#### Interventions

All subjects underwent an initial ophthalmic examination that included best corrected visual acuity, slit lamp, optometry, IOP, Pentacam, and fundus ophthalmoscopy. Based on the examination results, subjects who met the inclusion criteria were selected. Informed consent was obtained to ensure the compliance of subjects.

**Fig. 1:**
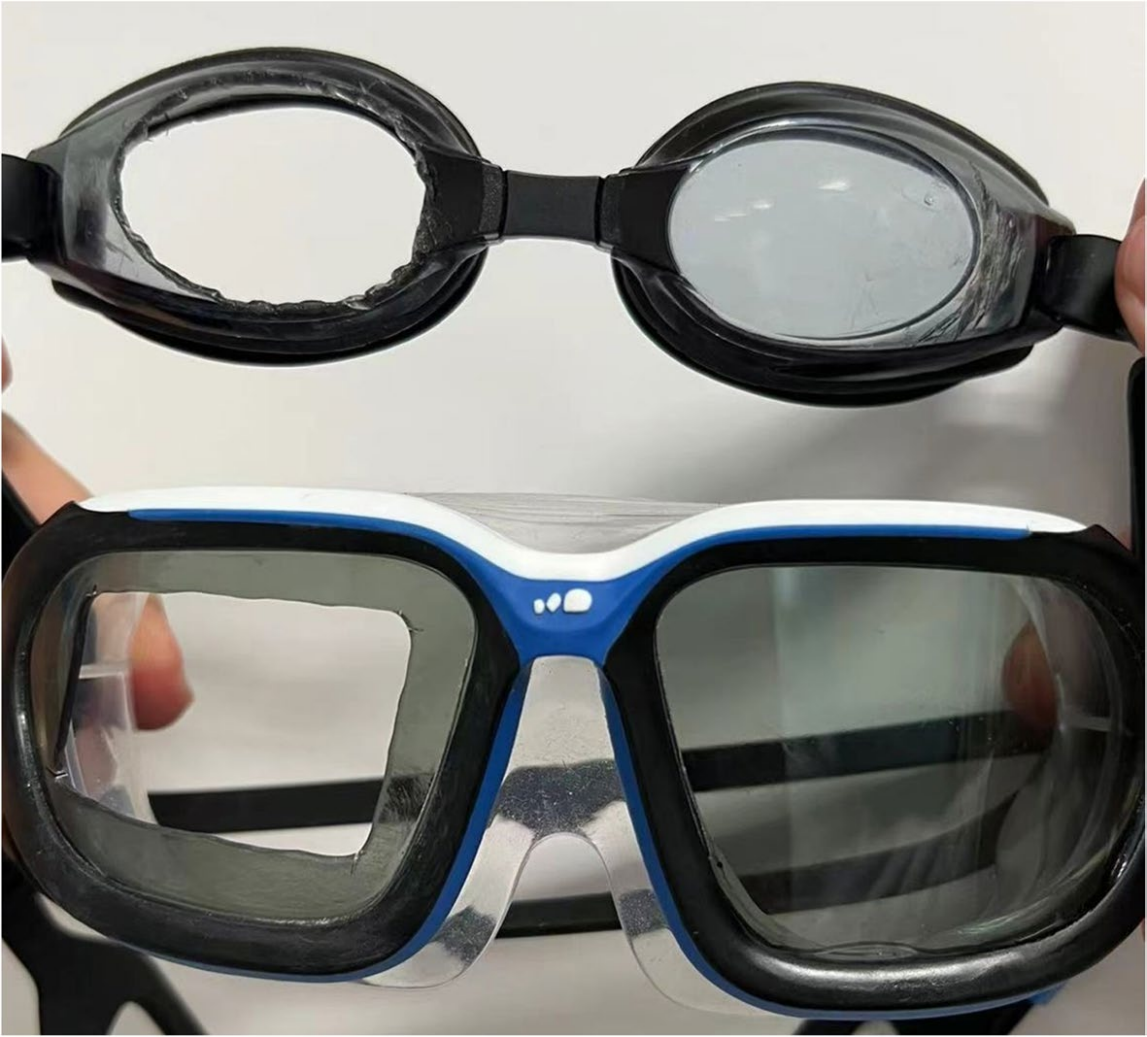
Eye-socket goggles SG1 (top) and wide vision swimming goggles SG2 (bottom), with the center part of the plastic eye mask for the right eye removed

The experiment was divided into two stages, in which the two types of eye socket goggles (SG1 and SG2) were worn respectively. Both goggle types were made by the same manufacturer (Li Ning, China), consisting of two separate rigid plastic eye cups, each sealed around the edge by rubber buffering, with an adjustable elastic band on either side. The center part of the plastic mask for the right eye was removed in both SGs for inspection and measurement, and the elastic bands of both SGs were adjusted to a fixed internal circumference that was 10 cm smaller than the circumference of the subject’s head to ensure that the elastic compression force of the SG was the same across all subjects. The SG1 cup measures 60 mm × 35 mm, and the SG2 cup 65 mm × 45 mm. Right eye data was collected from all subjects, as shown in Fig. 1.

#### Observation indicators

IOP and anterior segment parameters were assessed in relation to wearing either eye-socket or wide vision swimming goggles SG (SG1 and SG2, respectively). Subjects randomly drew lots to determine which of the two SGs were tested first, with a 10-min break between the two SGs tests. Each patient underwent Pentacam examination followed by non-contact tonometer examination. For data collection, participants were seated in front of the Pentacam and a contactless tonometer, while two experienced technicians evaluated the anterior segment parameters and IOP, performing rapid data collection.

The SG1 test was performed on the subjects by taking Pentacam and IOP readings at 5 time points (2 min before wearing, at 2 min and 5 min during wearing, immediately after removal, and 5 min after removal). After an interval of 10 min, the procedure was repeated with SG2. A total of 10 measurements were taken for each subject, 5 for each SG. The choice of measurement intervals was based on a study by Morgan et al. [1].

##### Intraocular pressure examination

The IOP examination was performed by taking the average of 3 consecutive measurements (in mmHg). Contactless tonometers have been shown to be affected by central corneal thickness (CCT ) [5]. In order to control for this influencing factor, all IOP data was corrected by the Pentacam using the Dresdner formula [6].

##### Pentacam check

The anterior segment parameters were obtained by the Pentacam high resolution rotating Scheimpflug camera (Oculus, Germany). This provided 3D anterior segment data and accurately measured the entire anterior segment from the anterior surface of the cornea to the posterior surface of the lens [7]. In this study, we performed a 25-picture scan and recorded the following criteria: flat axial corneal curvature (K1); steep axial corneal curvature (K2); average corneal curvature (Km); CCT; anterior chamber depth (ACD); anterior chamber angle (ACA), and; anterior chamber volume (ACV).

#### Statistical analysis

The normal distribution of the data (Shapiro-Wilk test) and the homogeneity of variances (Levene’s test) were first determined (*p* > 0.05). All values are presented as a mean ± standard deviation. Anova of repeated measurements was used for data involving repeated measurements (including the comparison of data at each time point before and after wearing SG), and independent sample T test was used for comparison between groups. For all analyses, an α value of .05 was adopted to define statistical significance and the Holm-Bonferroni correction was adopted for simultaneous comparisons.

## Results

### Subject demographics

A total of 22 females and 18 males were selected for this study, with the average age of the subjects being 28.7 ± 7.6 years. A total of 40 eyes (all right eyes) were analyzed, all subjects were Han nationality.

### Comparison of indicators before and after wearing goggles

#### Comparison of IOP

When wearing SG1, the IOP at both 2 min (21.0 ± 2.2 mmHg) and 5 min (21.2 ± 2.3 mmHg) was significantly higher than before wearing SG1 (17.7 ± 2.1 mmHg). Immediately after (18.4 ± 2.3 mmHg) and at 5 min after SG1 removal (17.7 ± 2.1 mmHg), the IOP decreased significantly (*P* <  0.001), but was still higher than before wearing the goggles (*P* = 0.009). The intraocular pressure recovered to baseline levels by 5 min after removal but was statistically significant (*P* <  0.001), as shown in Table 1.

**Table 1:** Comparison of intraocular pressure between the two groups

Indicators	Group	Baseline	2 min	5 min	After removing	Recovery
IOP (mmHg)	SG1	17.7 ± 2.1	21.0 ± 2.2	21.2 ± 2.3	18.4 ± 2.3	17.7 ± 2.1
	SG2	17.6 ± 2.1	17.8 ± 2.0	17.7 ± 2.0	17.6 ± 2.0	17.6 ± 1.9
	t	0.392	6.940	7.382	1.519	0.145
	P	0.696	< 0.001	< 0.001	0.133	0.885

There was no significant change in IOP during or after wearing SG2 (*P* = 0.931).

### Comparison of anterior segment parameters

The ACV(F = 44.363,p = 0.000), ACD(F = 27.451,*p* = 0.000), and ACA(F = 25.212, *p* = 0.000) values were smaller when wearing SG1. Compared with baseline at other time points, the ACV decreased when SG1 was worn (mean difference = 6.7, 8.7; *P* = 0.000, 0.000 2 and 5 min after SG1 was worn, respectively) and recovered when the SG1 was removed (mean difference = 1.3; *P* = 0.170). The baseline level was restored 5 min after removal (mean difference = − 0.600; *P* = 0.906). The ACD decreased when SG1 was worn (mean difference = 0.063, 0.077; *P* = 0.000, 0.000, 2 and 5 min after SG1 was worn, respectively), did not recover after SG1 was removed (mean difference = 0.021, *P* = 0.003), then returned to the baseline level 5 min after their removal (mean difference = − 0.002, *P* = 1.000). The ACA value noticeably decreased when SG1 was worn (mean difference = 0.940, 1.465, *P* = 0.000, 0.000, 2 min and 5 min after SG1 was worn, respectively), and had still not fully recovered even after SG1 was removed (mean difference = 0.403, *P* = 0.039). It later returned to the baseline level 5 min after SG1 was removed (mean difference = − 0.277, *P* = 0.339).

There was no statistically significant change in K1, K2, and Km when SG1 was worn compared with before, after, and 5 min after SG1 was removed.

Wearing SG2 did not cause any significant changes in the K1, K2, Km, CCT, ACV, ACD, and ICA values of the subjects, as shown in Table 2.

**Table 2:** Comparison of indicators before and after wearing two kinds of goggles (SG1 and SG2)

Indicators	Mirror	Baseline	2 min	5 min	After removing	Recovery
IOP (mmHg)	SG1	17.7 ± 2.1	21.0 ± 2.2	21.2 ± 2.3	18.4 ± 2.3	17.7 ± 2.1
	SG2	17.6 ± 2.1	17.8 ± 2.0	17.7 ± 2.0	17.6 ± 2.0	17.6 ± 1.9
K1 (D)	SG1	42.30 ± 1.20	42.35 ± 1.17	42.32 ± 1.18	42.32 ± 1.22	42.30 ± 1.19
	SG2	42.31 ± 1.20	42.30 ± 1.21	42.30 ± 1.19	42.31 ± 1.21	42.31 ± 1.19
K2 (D)	SG1	43.29 ± 1.20	43.32 ± 1.19	43.34 ± 1.18	43.29 ± 1.16	43.27 ± 1.16
	SG2	43.29 ± 1.19	43.31 ± 1.17	43.30 ± 1.21	43.29 ± 1.18	43.26 ± 1.17
Km (D)	SG1	43.29 ± 1.16	43.32 ± 1.13	43.34 ± 1.11	43.29 ± 1.13	43.27 ± 1.14
	SG2	43.28 ± 1.16	43.3 ± 1.17	43.30 ± 1.16	43.29 ± 1.22	43.26 ± 1.18
CCTS (mm)	SG1	536.6 ± 28.2	534.2 ± 29.4	533.9 ± 29.1	536.4 ± 27.6	536.2 ± 28.3
	SG2	536.3 ± 28.4	536.9 ± 27.7	536.5 ± 28.3	536.8 ± 28.4	537.0 ± 28.5
ACV (mm)	SG1	200.7 ± 33.6	193.0 ± 34.2	191.0 ± 32.7	198.4 ± 31.5	200.3 ± 33.5
	SG2	198.9 ± 33.1	199.2 ± 33.7	199.7 ± 34.1	198.4 ± 33.4	199.1 ± 33.7
ACD (mm)	SG1	3.14 ± 0.30	3.08 ± 0.29	3.07 ± 0.29	3.12 ± 0.29	3.15 ± 0.29
	SG2	3.14 ± 0.30	3.14 ± 0.30	3.13 ± 0.33	3.14 ± 0.30	3.15 ± 0.30
ACA (°)	SG1	40.84 ± 5.15	39.90 ± 5.39	39.37 ± 5.30	40.43 ± 5.67	41.11 ± 5.11
	SG2	40.90 ± 5.14	40.77 ± 5.25	40.85 ± 5.16	40.84 ± 5.20	40.98 ± 5.15

### Comparison of indicators when wearing different goggles for 2 min and 5 min

When wearing SG1 for 2 min and 5 min, the IOP of subjects was significantly higher than when wearing SG2, and the difference was statistically significant (2 min: t = 6.940, *P* <  0.001; 5 min: t = 7.382, *P* <  0.001). There was no significant difference in ACV, ACD, and ACA values between the two goggles, as shown in Tables 3 and 4.

**Table 3:** Comparison of each index between the two types of SG after 2 min of wearing

Grouping	IOPe (mmHg)	ACD (mm)	ACV (mm)	ACA (°)
SG1	21.0 ± 2.2	3.08 ± 0.29	193.0 ± 34.2	39.90 ± 5.39
SG2	17.8 ± 2.0	3.14 ± 0.30	199.2 ± 33.7	40.77 ± 5.25
t	6.940	0.904	0.819	0.732
P	< 0.001	0.369	0.415	0.466

**Table 4:** Comparison of each index between the two types of SG after 5 min of wearing

Grouping	IOP (mmHg)	ACD (mm)	ACV (mm)	ACA (°)
SG1	21.2 ± 2.3	3.07 ± 0.29	191.0 ± 32.7	39.37 ± 5.30
SG2	17.7 ± 2.0	3.13 ± 0.33	199.7 ± 34.1	40.85 ± 5.16
t	7.382	0.866	1.165	1.264
P	< 0.001	0.389	0.248	0.210

## Discussion

Wearing goggles while swimming or diving can protect eyes from water and prevent the onset of acute conjunctivitis, [8, 9] but the prevention of other diseases has not been reported. On the contrary, adverse reactions to wearing SG have been identified as follows: headache [10]; nasal deformity [11]; allergic dermatitis [12, 13]; different types of eye lesions, and; changes in intraocular pressure [14, 15]. A few studies have reported on the significant correlation between wearing goggles and intraocular pressure, with JesusVera et al. also finding that wearing goggles can reduce tear film rupture time and affect tear film stability. However, currently there are only a handful of reports on the influence of wearing goggles on the anterior segment [16]. Raimundo et al. showed that corneal thinning (54.8 ± 41.1 μm), atrial angle narrowing (2.6 ± 2.6°) and an increase in intraocular pressure (4.0 ± 1.9 mmHg) when wearing goggles had no effect on anterior chamber volume [3]. Also, Dondu Melek Ulusoy et al. showed no significant change in anterior segment measurements, with the exception of ACV, while wearing goggles in their study of keratoconus patients, and concluded that the short-term use of goggles does not increase the risk of corneal parameter deterioration in this population [4].

The results of this study showed that IOP increased significantly in the short term, while ACV, ACD, and ACA decreased, with all returning to the baseline level immediately after the goggles were removed. Corneal curvature, CCT, and other indicators showed no significant change. These findings may have implications for the prevention of a variety of eye diseases, as the increase and fluctuation of IOP is a potential risk factor for the occurrence and development of glaucoma, as well as an influential factor in early refractive regression after corneal refractive surgery.

Our results are consistent with those of Raimundo et al.*,*[3] which also show an increase in intraocular pressure after wearing SG. However, contrary to our results, Raimundo et al. showed that CCT decreased by 55 μm while SG was worn. Both values returned to the pre-wear level after SG removal. The researchers concluded that SGs exert mechanical pressure on the orbital tissue and compress the eyeball, leading to significant thinning of the cornea. This discrepancy with our results may be related to the difference in the orbital margin area caused by ethnic differences.

In addition to verifying our hypothesis that wearing eye socket type goggles results in higher intraocular pressure, this research is novelin that it shows that SGs with different size and design not only exert different levels of compression force on the eye and orbital tissue, but also have different degrees of influence on the biological characteristics of the anterior eye.

Drawing on the experimental design of previous studies, we removed the front part of the eyecup of each SG to assess both IOP and anterior segment morphology while they were being worn. Therefore, our experimental design did not accurately replicate realistic conditions, resulting in relatively conservative results. In addition, we also drilled into the face of the goggles, which caused a loss of airtightness and eliminated the suction effect. Under realistic conditions, the appropriate SG suction is 0 to 25 MMHG. The effect is equivalent to the feeling created by an elevation of 60 m and is not expected to affect eye indicators including IOP. Our measurements also do not include the increase in pressure of 0.74 mmHg per centimeter of water depth that affects the external water pressure on the goggles [1]. As such, it can be expected that unmodified SG, combined with the effect of external water pressure, may contribute to a larger increase in IOP. In addition, all measurements were taken in the absence of physical activity, and it is important to consider that IOP and anterior segment parameters may vary based on exercise conditions and may also depend on a number of factors, such as exercise intensity, the participant’s fitness level, and the time of measurement [17, 18]. A potential solution is to use Triggerfish (Sensimed), a new eye pressure sensing contact lens, to assess changes in IOP during swimming [19]. Secondly, the subjects of this study were chosen by random lottery to determine the order of the two SG tests. There was a 10 min rest between the two SG test times. As shown in previous studies, when the SG are removed, the intraocular pressure can return within a very short time to baseline levels. However, the two SG before and after the test are still likely to interfere with this, especially after the first round of experimental results from the SG1 and SG2 tests. Thirdly,our study was carried out with healthy subjects, so our results cannot be directly applied to patients, such as those with glaucoma, keratoconus, or who have undergone corneal refractive surgery. Finally, the current study is limited to the short-term effects of wearing SG. While a recent study showed that adult swimmers who used SG regularly did not have a higher incidence of glaucoma compared to non-swimmers, further research is needed on the possible long-term effects of wearing SG on eye health [20]. Despite the above limitations of this study, we believe that, depending on individual differences, some subjects are more sensitive than others to such factors affecting IOP, and the current findings may provide some guidance for the long-term care of some ophthalmic diseases and postoperative patients, especially those who are accustomed to swimming. However, according to our results, the use of eye socket type SG in swimming or water sports may produce negative effects on eye health, especially in patients with eye disease. Thus, we suggest that water sports enthusiasts, especially corneal refractive surgery patients and patients with glaucoma, consider wearing the larger framed orbital SG as opposed to ocular socket SG.

## Supplementary Information

**Additional file 1.**

## Data Availability

All data generated or analysed during this study are included in this published article.

## Abbreviations

IOP: Intraocular pressure
SG: Swimming goggles
K1: Flat axial corneal curvature
K2: Steep axial corneal curvature
Km: Average corneal curvature
CCT: Central corneal thickness
ACD: Anterior chamber depth
ACA: Anterior chamber angle
ACV: Anterior chamber volume
